# SIMPD: an algorithm for generating simulated time splits for validating machine learning approaches

**DOI:** 10.1186/s13321-023-00787-9

**Published:** 2023-12-11

**Authors:** Gregory A. Landrum, Maximilian Beckers, Jessica Lanini, Nadine Schneider, Nikolaus Stiefl, Sereina Riniker

**Affiliations:** 1 Department of Chemistry and Applied Biosciences, ETH Zurich, Vladimir-Prelog-Weg 2, Zurich, 8093 Switzerland; 2 Novartis Institutes for BioMedical Research, Novartis Pharma AG, Novartis Campus, Basel, 4002 Switzerland; 3 Present Address: F. Hoffman-LaRoche AG, Grenzacherstrasse 124, 4070 Basel, Switzerland

**Keywords:** Lead optimization, Cross-validation, Machine learning

## Abstract

Time-split cross-validation is broadly recognized as the gold standard for validating predictive models intended for use in medicinal chemistry projects. Unfortunately this type of data is not broadly available outside of large pharmaceutical research organizations. Here we introduce the SIMPD (simulated medicinal chemistry project data) algorithm to split public data sets into training and test sets that mimic the differences observed in real-world medicinal chemistry project data sets. SIMPD uses a multi-objective genetic algorithm with objectives derived from an extensive analysis of the differences between early and late compounds in more than 130 lead-optimization projects run within the Novartis Institutes for BioMedical Research. Applying SIMPD to the real-world data sets produced training/test splits which more accurately reflect the differences in properties and machine-learning performance observed for temporal splits than other standard approaches like random or neighbor splits. We applied the SIMPD algorithm to bioactivity data extracted from ChEMBL and created 99 public data sets which can be used for validating machine-learning models intended for use in the setting of a medicinal chemistry project. The SIMPD code and simulated data sets are available under open-source/open-data licenses at github.com/rinikerlab/molecular_time_series.

**Supplementary Information:**

The online version contains supplementary material available at 10.1186/s13321-023-00787-9.

## Introduction

Validating the performance of a new machine learning (ML) approach or descriptor requires a large collection of data sets which are reflective of the types of data the method will eventually be applied to. For methods intended for use in the context of medicinal chemistry projects, the gold standard for model validation is to use data from other medicinal chemistry projects and to split the data into training and test sets based on the order in which the compounds were actually made or tested. This approach, known as time-split cross-validation [1], tests models the way that they are intended to be used. Despite the name, the important factor here is not the time itself, but the ordering of the compounds into early (training) and late (test) sets. This recognizes that the compounds made and tested later in a medicinal chemistry project are typically designed (or selected) based on the knowledge derived from data obtained by testing earlier project compounds. This “continuity of design” is a key feature of lead-optimization data sets.

Unfortunately, time-split cross-validation is not possible when working with public data sets, where we normally do not have access to large sets of data generated within a single project and where information about the order in which compounds were made and tested is typically not available. Although resources like ChEMBL [2] do include dates for many of the scientific publications from which data was curated, these publications are often from different research groups and/or projects and thus lack the design connection between early and late compounds. Common alternatives typically rely on randomly splitting the data set or using some form of chemical information (like fingerprints or scaffolds) to construct training/test splits (termed neighbor splits in the following). These methods have well-known shortcomings: the random approaches tend to overestimate model performance and the neighbor splits tend to be overly pessimistic about model accuracy (see Figure 2 in Ref. [1]). Both of these failure modes can lead us astray when assessing the utility of a new method: overly optimistic validation results make us use a model which does not perform well when used prospectively, while overly pessimistic results can lead to the rejection of a model which may actually be useful.

We focus here on project-specific assay data from medicinal chemistry projects (i.e., no service assays for e.g. ADMET). This type of data mainly comprises biochemical and cellular assays that measure potency/efficacy against a target or an off-target and usually contains a strong shift over time in terms of both chemical matter (the independent variable) and measured values (the dependent variable). The more that is known about a target, the better the design of the compounds get over time. Typically, this leads to an overall trend of increasing potency from the earliest to the most recent compounds. However, as other properties are optimized at the same time as activities, project teams will sometimes accept minor trade-offs on activity during optimization. The size of these data sets is usually relatively small, comprising tens to low thousands of measured values. All of these characteristics make project data sets challenging to model with ML methods. It is also important to note that the limited chemical diversity of project data sets means that models built upon them are generally only applicable to related compounds. These data sets typically do not produce models for virtual screening of diverse chemical collections, however, they can be useful for virtual screening of focused chemical spaces which are chemically related to what a team knows already. The other important properties mentioned above for compounds in medicinal chemistry projects, like physico-chemical and ADME properties, are typically measured in so called “service assays”. Such assays are generally used across medicinal chemistry projects to profile compounds, and therefore consist of a much larger number of more diverse compounds. Data sets from service assays do not show the pronounced time-dependent trends in properties seen for project assays and can thus be more reliably modeled assuming a random distribution. For this reason, we do not consider service assays in this study.

**Fig. 1:**
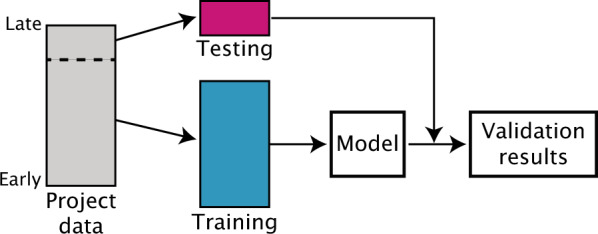
Schematic of the process of time-split cross -validation

In this study we introduce a method to divide data sets into training/test sets that differ in ways similar to what is observed in temporal splits of real-world medicinal chemistry project data (Fig. 1). We start by curating a large collection of data sets from small-molecule drug discovery projects run within the Novartis Institutes for Biomedical Research (NIBR) in order to identify a small number of properties which consistently change in the same way between the early and late phases of the projects. These properties are used as objectives in a (multi-objective) genetic algorithm that can be applied to new data sets to generate training/test splits with similar property differences. We validate the approach, which we call simulated medicinal chemistry project data (SIMPD), by demonstrating that the splits it generates for the NIBR project data sets do mimic the real-world time splits and that they are, in general, more predictive of temporal split ML performance than either random or neighbor splits. Finally, we apply SIMPD to data drawn from ChEMBL in order to create a collection of open data sets with simulated time splits. We anticipate that the data sets constructed using the SIMPD algorithm will be useful for benchmarking the utility of new ML approaches or descriptors to building project-specific models.

## Methods

### Curating medicinal chemistry project data

Project assays were extracted from an internal NIBR data set of bioactivity assays that had already been curated and cleaned. Only assays from terminated or completed projects were used. Repeat measurements of the same assay on the same compounds were combined using the arithmetic mean of the pAC50 values. As we are focusing in this work on data sets from single projects, we removed assays where no clear assignment to a specific internal project could be achieved (i.e., assays without a project code) as well as those with $> 10,000$ compounds (these can be project assays that became service or panel assays). In addition, we removed assays with $< 200$ compounds, as these were likely only measured sporadically in the respective projects and only provide limited information.

Compounds with high variability of the activity measurement (standard deviation $> 0.1*$mean pAC50) were removed from the data set, as the corresponding measurements are likely unreliable. Furthermore, only molecules with a molecular weight between 250 and 700 g/mol are used. The NIBR substructure filters [3] were applied to remove unwanted molecules (small peptides, macrocycles, etc). Similarly, we only kept compounds with a fraction of nitrogen and oxygen $>0.0$ and $<0.6$.

Over the course of a drug discovery project it is not uncommon that compounds which were not explicitly synthesized for a project will be tested in the project assays. To reduce the chances of having these unrelated “extra” compounds appear in our data sets, we determined the main time period of the assay. Given the sample registration dates of the compounds, all years with $> 50$ compounds were identified and the beginning/end of the main period were defined to be the first/last of these dates. Only compounds registered during this main period were kept for each assay.

The set of assays was further pruned by removing all assays with a pAC50 range smaller than three log units. To enable the use of classification models, we used a pAC50 threshold of 6.3 to group compounds into active and inactive sets (inactive: $< 6.3$, active: $\ge 6.3$). Based on this classification, we removed all assays with active or inactive ratios $< 0.05$ in either the entire data set or either the earliest 80 % or latest 20 % of compounds (based on registration date). To focus on assays from lead-optimization projects, we removed those that do not contain any pharmacokinetic data associated with the compounds as well as those where less than 25 % of all measured compounds were registered with the project code of the corresponding assay.

Finally, we wanted to remove highly correlated assays from the set, i.e., assays with similar compounds and measurement results. Using the compound IDs together with their active-inactive classifications, we clustered the set of assays for each assay project code using a Jaccard similarity threshold of 0.5. From these clusters, we then only keep the assay with the most measurements.

### Temporal, random, and neighbor splitting

A subset of the available data is usually used to train and optimize the hyperparameters of bioactivity models with the rest of the data being used to assess their performance. Such a partition, as previously explained, can be done in different ways. In this work three main splitting strategies are considered, together with the proposed SIMPD: temporal, random, and neighbor splits.

Temporal splits are constructed by ordering the data according to the date on which the measurements have been made in ascending order and using the first X % of the data for training the models and the last 100-X % for testing. In this way, one can simulate the process of prospective validation and assess the model performances in the most realistic manner.

Random splitting is realized by randomly selecting a fraction of the compounds for training and using the rest to test the models. The split is done in a stratified manner based on the activity class. Thus, the training and test sets both contain the same ratio of active to inactive compounds. To reduce any accidental performance bias given by the selection of a specific random test/training set, ten different random seeds were used to create ten different random splits. The final performance values are provided as medians and standard deviations over the ten repeats.

Neighbor splits were obtained by ordering the data set according to the decreasing number of neighbors each molecule in the data set has and then using the first X % for training and the last 100-X % for testing. In our set-up, two compounds are considered neighbors if their Tanimoto similarity based on the Morgan fingerprint [4] as implemented in the RDKit v2021.09.5 [5] with radius 2 is $\ge 0.55$. For this type of split, it is possible that the compounds around the train/test separation threshold have the same number of neighbors, but they fall into the two different sets. In this case, one can randomly rearrange them according to the different seeds, obtaining slightly different training and test sets. Here, ten different seeds were used.

In this work, all training/test splits were done 80/20, i.e., 80 % of the data was used for training and the remaining 20 % for testing.

### Building models for bioactivity

To build models for bioactivity, Morgan fingerprints [4] with radius 2 were used as features and the random forest classifier was selected as the estimator. Random forests [6] were built using the implementation in scikit-learn, version 0.24.2 [7].

The hyperparameters optimized for the random forest include *max_depth*, *min_samples_leaf*, and *n_estimators*. Two possible values were used for each of these: [20, 40], [2, 4], and [100, 300], respectively. A nested grid-search cross-validation was used to identify the best set of hyperparameter values. In this case, five folds and two folds were used for the outer and the inner loop, respectively. The set of hyperparameter values that was selected most often among the five folds was the one used to train the final model on the entire training set. The instability of the model was identified by considering the cases where five different sets of hyperparameter values were selected in the outer loop. None of the models built here was flagged as *instable* by this procedure.

The training set used for hyperparameter optimization was obtained by different splitting strategies, e.g., random, temporal, neighbor, and the SIMPD algorithm. When ten different random number seeds were used to split the original data set into ten test/train sets, only the first one was taken to determine the best hyperparameters. The other nine partitions employed this hyperparameter set. Because some of the data sets are imbalanced, the probability threshold was adjusted for each classifier by using the ghostml library [8]. The adjusted thresholds were selected to optimize Cohen’s $\kappa$ score [9] on the training data.

The evaluation of classifier quality is based on different metrics: balanced accuracy, F1 score, precision, recall, and Cohen’s $\kappa$ score [9]. These metrics are calculated using scikit-learn, version 0.24.2 [7]. For each metric, the lists of the means and standard deviation values (calculated over the ten repeats) were collected for each splitting strategy. Since there is only one temporal split per data set, the corresponding standard deviation here was set to zero.

The process of computing the best random forest classifier for each assay, each split and eventually, each seed, was fully automated using the PREFER library [10]. An 80 % / 20 % training / test split was used for all splitting strategies.

### Evaluating the differences between molecules in training-test splits

#### Descriptor-based methods

Our goal was to identify a small set of non-correlated, ideally interpretable descriptors whose distributions consistently change over the course of medicinal chemistry projects. A set of 418 descriptors was generated for all compounds. The descriptor set consists of 209 standard RDKit descriptors (see the Additional file 2 for the complete list) in both their standard and “normalized” (i.e., divided by 1000 times the number of heavy atoms in the molecule) forms.

The distributions of each descriptor in the sets of early and late compounds were compared using the Brunner-Munzel test (as implemented in scipy, version 1.8.1 [11]). Only descriptors with a p-value $< 0.01$ were considered for further analysis. We ranked the descriptors based on how consistent the direction of the shift in their median values between early and late compounds was across all projects. From the top ranked ones, we manually picked a small set of interpretable descriptors whose values are not strongly correlated with each other according to the Spearman rank-order correlation coefficient (as implemented in scipy, version 1.8.1 [11]).

#### Similarity-based methods

To get an unbiased view of the chemical changes between the early (training) and late (test) sets of compounds, we applied a variation of the refined nearest neighbor analysis first used for chemical problems by Rohrer and Baumann [12]. This approach is driven by the cumulative probability distribution functions (CDF) of the distances between nearest-neighbor compounds in two data sets. We use two different functions to characterize the data sets: *G*(*t*), which is the CDF for distances from compounds in the late (test) set to nearest neighbors in the late (test) set, and $F'(t)$, which is the CDF for distances from compounds in the late (test) set to nearest neighbors in the early (training) set. So *G*(*t*) is generated by finding the distance between each compound in the late (test) set and its nearest neighbor in the late (test) set and then plotting the fraction of compounds with a neighbor distance < a threshold *t* as a function of *t*. $F'(t)$ is analogous but the distances are between compounds in the late (test) set and their neighbors in the early (training) set. Note that the definition of $F'(t)$ differs from Rohrer and Baumann’s *F*(*t*) [12], which considers distances from a bootstrap sample of early and late compounds to their nearest neighbors in the late (test) set. Finally, we also generated $S'(t)$, which is defined to be $G(t)-F'(t)$.

The *G*(*t*), $F'(t)$, and $S'(t)$ curves can all be usefully summarized by summing the values on a grid of possible *t* values. Large values of $\sum G(t)$ indicate clustering/clumping in the test set, while large $\sum F'(t)$ values occur for data sets where the compounds in the test set all have close neighbors in the training set. Positive/negative $\sum S'(t)$ values indicate that the test set compounds are closer to/farther from the training set compounds than they are to each other, with the magnitude of $\sum S'(t)$ showing the degree to which this is true.

In this work, $\sum G(t)$, $\sum F'(t)$, and $\sum S'(t)$ were all calculated with *t* values on a grid from 0.0$-$1.0 in 100 steps.

### SIMPD algorithm

The idea of the simulated medicinal chemistry project data (SIMPD) algorithm is to use a multi-objective genetic algorithm (MOGA) to split a set of compounds with bioactivity data into one or more training and test sets that differ from each other in ways resembling the differences between the temporal training/test splits observed in medicinal chemistry projects. The key steps are:

1. Choose starting populations for the MOGA
2. Choose objectives to be used in the MOGA
3. Choose constraints to be used in the MOGA
4. Run the MOGA to generate a set of solutions
5. Rank the solutions from the MOGA and select one or more to be used for model building and validation

Each of these is discussed in detail below.

#### Choosing starting populations

The MOGA requires an initial population of solutions, which are derived from the full data set. Here, the initial populations were chosen by clustering the full data set (see below) and then randomly assigning clusters to the test set until the desired number of compounds was present in the test set. If the assignment of a cluster to the test set would make the test set too large, a random subset of the cluster containing only the required number of compounds was added. The rest of the compounds from that cluster remained in the training set.

To start from reasonably sized initial clusters and have a fully automated procedure, we performed a Taylor-Butina clustering [13, 14] of the compounds in the data set using Morgan fingerprints with a radius of 3 and a similarity threshold of 0.35. The clustering was done using the RDKit’s implementation with the reordering option enabled. Since Taylor-Butina clustering can yield a large number of singletons, we combined singletons and compounds from small clusters (size $< 2$ % of the compounds) into larger clusters. Compounds were merged into the closest large cluster, where the compound–cluster distance was defined to be the minimum distance between the compound to be merged and a compound in the cluster.

#### Objectives

Based upon analysis of descriptor differences and the spatial statistics between training and test sets in the NIBR medicinal chemistry projects (see Results and Discussion section), we selected eight objectives for the MOGA:

1. $$\Delta _{\text {test-train}}$$ median(SA_Score) = 0.28
2. $$\Delta _{\text {test-train}}$$ median(HeavyAtomCount) = 3.1
3. $$\Delta _{\text {test-train}}$$ median(TPSA) = 13.2
4. $\Delta _{\text {test-train}}$ median(fr_benzene/1000 HeavyAtoms) = $-$8.8
5. frac$_{\text {active}}$(train) = value from data set
6. frac$_{\text {active}}$(test) = value from data set
7. $$10< \sum G - \sum F' < 30$$
8. $$\sum G > 70$$

The target values for objectives 1–4, 7, and 8 were the same for every data set (and the source of these values is discussed below in the Results and Discussion section), while the targets for objectives 5 and 6 were determined by the temporal splits of the original data set. Definitions of, and references for, the descriptors used for the first four objectives are also below in the Results and Discussion section.

#### Constraints

The optimization used two constraints, the first to ensure that the test set has the desired size and the second to ensure that at least some of the clusters survive:

1. The number of compounds in the test set must be 20 % of the total number of compounds in the data set.
2. The relative cluster population entropy of the test set must be $< 0.9$.

Relative cluster population entropy was determined by dividing the information entropy [15] of the cluster populations by $log_2(\text {number of clusters})$ (i.e., the maximum possible information entropy for the number of clusters). The values range from 0.0 (i.e., all test set compounds are in a single cluster) to 1.0 (i.e., the test set compounds are evenly distributed across the clusters).

#### Running the genetic algorithm

We performed the MOGA using the NSGA2 algorithm [16] as implemented in the package pymoo, version 0.50 [17]. The optimization was done using binary crossover and a mutation operator that randomly replaces 10 % of the test set with compounds from the training set. For each data set, the GA was run for 300 generations from a starting population of 500 members.

#### Selecting solutions

The MOGA provides a set of up to 500 solutions, each of which are located on the Pareto front for the eight objectives. As working with 500 versions of each data set is impractical, we used a simple strategy to rank the solutions and select a single one. Since each solution from the NSGA2 optimization is Pareto optimal, we do not have any quality metric directly available from the GA itself to rank the solutions. Therefore, we chose to score how well each objective was satisfied and then to generate a score for the solution based on a weighted sum of the objective scores,

1 $$\begin{aligned} \text {score}_i = \sum _{j=1}^{8} w_j * e^{-x_{ij}}, \end{aligned}$$

where the weights ($w_j$) are 10 for the $\sum F'(t) - \sum G(t)$ objective, 5 for the $\sum G(t)$ objective, and 1 for the others, and the $x_{ij}$ are the scaled objective values for solution *i*. Since the objectives themselves have different magnitudes, each objective value was scaled such that its value is 1 at the 90th percentile of the values observed across all 500 populations. The solution with the highest score from Eq. 1 was used.

In this weighting scheme, we have chosen to favor solutions that better satisfy the two objectives derived from spatial statistics. This reflects our hypothesis that the relationship of the chemical similarities within the test set and between the training and test sets are an essential part of what makes temporal data splits from medicinal chemistry project data different from random or neighbor splits.

### Validation of the SIMPD algorithm

We validated the SIMPD algorithm by applying it to the NIBR medicinal chemistry project data sets and asking two questions:

1. Do the differences between the training and test compounds in the SIMPD splits look similar to those in the actual temporal splits?
2. Do the SIMPD splits provide a better estimate of the time-split based ML performance than the random or neighbor splits?

The first question is straightforward to answer by looking at how well the simulated time splits from SIMPD reproduce the trends from the project data sets which were used as objectives in the MOGA.

Our primary motivation for generating simulated time splits with SIMPD is to be able to predict the performance of a predictive model when applied prospectively to project data. The performance of an ML model trained and validated using SIMPD splits should be more similar to the performance of a model built using a temporal split than models built with either random or neighbor splits are. To do this evaluation and answer the second question, we applied the ML setup described above to the SIMPD data sets and compared the area under the ROC curve (AUC) values of the different approaches.

### Selecting public data sets

We curated a collection of baseline bioactivity data sets from ChEMBL32 [2]. Our goal was to have a collection of data sets for the SIMPD algorithm that are similar to what we observed in the NIBR medicinal chemistry project data. This means that we need a reasonable range of activity values and a good degree of clustering in the data.

To avoid the potential pitfalls and noise associated with combining data from different ChEMBL assays, we limited ourselves to measured K$_\text {i}$ data sets associated with publications where pChEMBL values have been assigned [18] and with measurements for 300–1000 unique compounds for any given target. The focus on K$_\text {i}$ data sets with a date and the upper limit of 1000 unique compounds were imposed to exclude screening assays. To ensure that the data sets are maximally consistent, we were very strict in our curation of the K$_\text {i}$ assays which would be combined for a given data set and only combined assays with consistent values for target id, assay organism, assay category, and BAO format. The full details of the 99 data sets in ChEMBL32 that fulfilled these criteria are given in the Additional file 2.

**Fig. 2:**
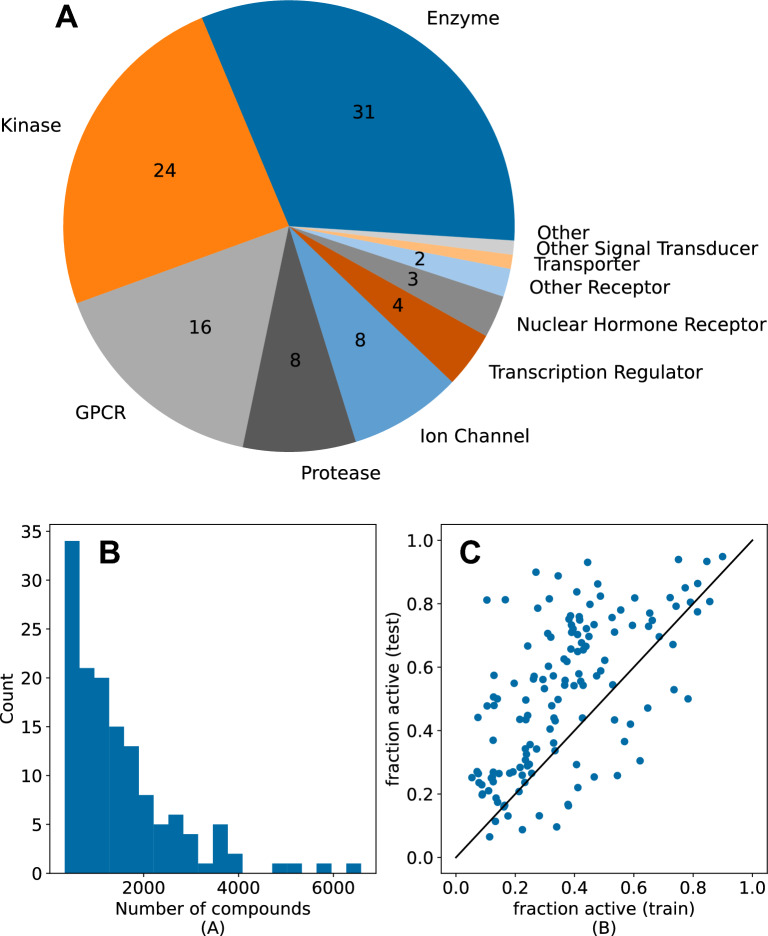
Composition of the NIBR medicinal chemistry project data sets. **A**: Target classes for the assays. The black numbers denote the number of the 138 assays with this target class. 39 of the assays do not have a target class assigned. **B**: Histogram of data set sizes. **C**: Scatter plot of the fraction of active compounds in the test set versus the fraction of active compounds in the training set based on the temporal splits

In order to assign active/inactive labels to the data, we picked a pChEMBL threshold for each assay which led to a 60/40 inactive/active split – a value that is consistent with our observations from the NIBR project data sets (Fig. 2).

### Applying SIMPD to public data sets

Once the SIMPD algorithm was validated using the NIBR data sets, we applied it to the 99 public bioactivity data sets from ChEMBL32 in order to generate an initial collection of diverse data sets that can be used to benchmark ML algorithms.

The temporal splits for the medicinal chemistry project data sets provided the target values for $\text {frac}_{\text {active}}$(train) and $\text {frac}_{\text {active}}$(test) in the SIMPD algorithm. When creating simulated splits from public data sets, however, these values are not available, so target values have to be selected. Rather than optimizing $\text {frac}_{\text {active}}$(train) and $\text {frac}_{\text {active}}$(test) independently, we decided to optimize the single objective $\Delta \text {frac}_{\text {active}}$(test - train) for the public data sets: the 50th and 90th percentile values of $\Delta \text {frac}_{\text {active}}$(test - train) from the NIBR project data sets (see Additional file 1: Fig. S1). As the results for these two different values did not differ qualitatively from each other (data not shown), we present only the results for the median (50th percentile) value of $\Delta \text {frac}_{\text {active}}$(test - train) = 0.11 in the following. The specific objective for the GA to minimize here was thus $| \Delta \text {frac}_{\text {active}}(\text {test} -\text {train}) - 0.11|$.

## Results and discussion

### Summary of the project data sets

#### Overview

The curation of the NIBR medicinal chemistry data sets led to a set of 138 different project data sets, containing a total of 207,172 different assay measurements and 157,885 unique compounds. Figure 2B shows the distribution of the sizes of the individual project data sets. Most of the data sets are small (in an ML context) and consist of < 1000 different compounds (42 % of the data set) with the smallest containing 335 molecules and the largest 6585 compounds. The time-span of these project data sets ranges from 2.5 up to 15 years with an average of 5.7 years. Several different target classes are included with the most common being enzymes (31), followed by kinases (24) and GPCRs (16), a detailed distribution can be found in Fig. 2A. As discussed above, this set of assays does not include service assays like those used to measure ADME/toxicity or physical properties like solubility.

The 138 data sets have measured pAC50 values that stem from biochemical as well as cellular assays with a mean range of 4.7 log units. To enable classification models, we used a threshold of pAC50 = 6.3 (500 nM) to label compounds as being active or inactive (see Methods section). We used the timestamp information to split our data sets into training and test sets (20 % of the most recent data) as described above. This can lead to a different fraction of active compounds in the training and test sets (Fig. 2C) and might cause inverse imbalance ratios (actives versus inactives) between training and test set. This is a major difference between public and typical project data sets [8]. Overall, all data sets have a reasonable fraction of actives in the training and test sets ranging from approximately 10 % up to 90 % (Fig. 2C). For the data sets with a very high fraction of actives (upper right corner in Fig. 2C), a stricter activity threshold may have been more appropriate. Only a few data sets have the same distribution of actives in training and test set (diagonal in Fig. 2C). As expected for late-stage medicinal chemistry projects (i.e., the structure-activity relationships are well studied), most of the data sets show a higher fraction of actives in the test set (the latest compounds).

#### Machine-learning performance

**Fig. 3:**
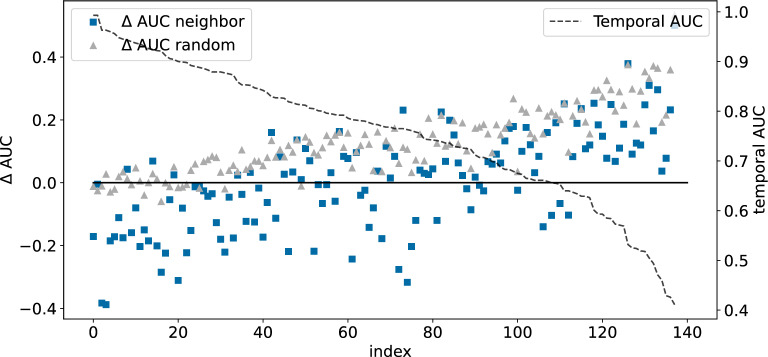
Validation performance of random forest models on the NIBR medicinal chemistry project data sets. The black dashed line and right hand *y*-axis show the AUC values for the temporal splits. The gray triangles and blue squares show $\Delta$ AUC values (left hand *y*-axis) for the random and neighbor splits, respectively. The points are ordered by decreasing temporal AUC values

In Ref. [1], regression models built on random/neighbor splits were found to be generally more/less accurate than the models built on temporal splits of the same data sets. To see if this is also the case for classification models built on the NIBR medicinal chemistry project data sets, we compared the AUC values for the three different split types in Fig. 3. To facilitate direct comparison, the AUC values for the temporal splits are presented as a dashed line (the right axis), while the $\Delta$AUCs relative to the temporal splits for the neighbor and random splits are plotted with squares and triangles (left axis). The data sets have been ordered by decreasing AUC of the temporal splits. These data clearly demonstrate that random splits tend to be more “optimistic” about model performance than neighbor splits. They generally have $\Delta$AUC values which are positive and larger than the corresponding value for the neighbor split.

It is important to notice in Fig. 3 that there are data sets for which $\Delta$AUC is positive for *both* random and neighbor splits. This is a trivial result for data sets at the far right-hand side of the plot, where the AUC values for the temporal split are very low, but non-obvious, and perhaps surprising, for the data sets in the middle and left-hand side of the plot where temporal AUC values exceed 0.65 or 0.70. This observation highlights that the conventional wisdom of neighbor splits leading to overly pessimistic estimates of model quality does not always hold for real-world data sets.

### Descriptor and similarity differences in the temporal splits

The final set of descriptors selected consists of the “synthetic accessibility” score (SA_Score) [19], heavy atom count, topological polar surface area (TPSA) [20], and the number of benzene rings per 1000 heavy atoms (fr_benzene/1000 HeavyAtoms), see Table 1. The first three of these tend to increase over the course of a project while the frequency of benzene rings tends to decrease. These trends match what we expect to observe in medicinal chemistry projects: As the project progresses, compounds tend to get more complex/difficult to synthesize (higher SA scores indicate compounds which are harder to synthesize), larger, and more polar (this is reflected both in the increase in TPSA as well as the decreasing frequency of benzene rings). These trends are not surprising based upon other studies of this type (see e.g., Ref. [21] and references therein).

**Table 1:** Summary of the distribution changes for the descriptors chosen

Property	Sign	Frac	Median (frac change)	Median (train)	Number of projects
SA score	1	0.88	0.09	2.8	109
HeavyAtomCount	1	0.75	0.09	31.0	114
TPSA	1	0.76	0.14	88.6	109
fr_benzene/1000 HeavyAtoms	-1	0.81	0.19	43.5	118

The meanings of the columns are: **Sign** = sign of the difference between training and test; **Frac** = fraction of the data sets showing a change with that sign; **Median(frac change)** = median fractional change of the value across the data sets; **Median(train)** = median value of the property in the training set; **Number of projects** = number of projects where the difference in the training/test distributions was statistically significant (see text)

**Fig. 4:**
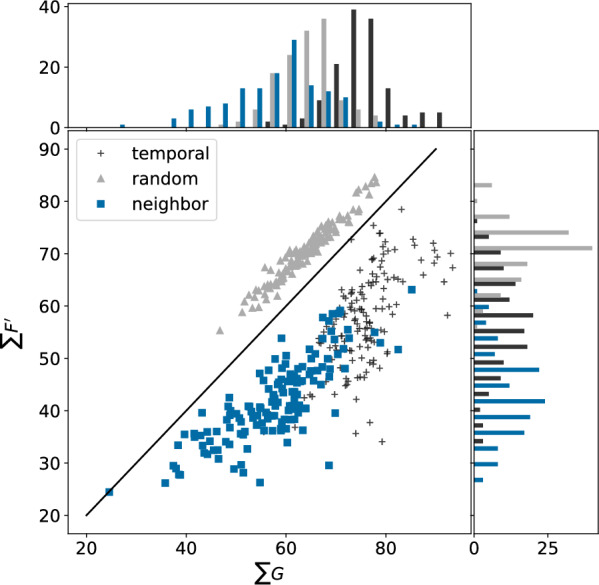
Spatial statistics summary plot $\sum G$ against $\sum F'$ for the NIBR medicinal chemistry project data sets with temporal (black crosses), random (gray triangles), and neighbor (blue squares) training/test splits

A summary of the $\sum G$, $\sum F'$ values for the NIBR medicinal chemistry project data sets is shown in Fig. 4. The full spatial statistics curves for all of the data sets are provided in Additional file 1: Fig. S2. The points from the three different splitting strategies are reasonably distinct in the scatter plot in Fig. 4, with the random split points above the diagonal (comparatively higher $\sum F'$). There is some overlap between the neighbor and temporal splits, which both appear below the diagonal (comparatively higher $\sum G$), but the temporal splits tend to be shifted to the upper right (higher values of both $\sum F'$ and $\sum G$).

We would expect the points for random splits to lie above the diagonal in Fig. 4, indicating that test compounds have closer neighbors in the larger training set than they do in the smaller test set, since any given compound is more likely to have a close neighbor in a larger set than a smaller one when the division is done randomly. The converse of this argument applies to the neighbor splits, where compounds are selected to be in the test set because they have few neighbors in the overall data set. Lying below the diagonal in Fig. 4 where $\sum G > \sum F'$ also implies that $\sum S'$ should be negative. This can be seen in the $S'$ curves in Additional file 1: Fig. S2.

While the location of the points with the random and neighbor splits in Fig. 4 can be rationalized, it is not possible *a priori* to predict location of the temporal splits. The observed behavior, that the points for the temporal split lie below the diagonal, indicates that the test (later) compounds are more likely to have close neighbors in the later compounds than they are in the earlier compounds. The distribution of $\sum G$ values indicates that compounds in the test sets for the temporal splits are more similar to each other than what is observed in either the random or neighbor splits.

### Validation of SIMPD using project data

#### MOGA objectives

**Fig. 5:**
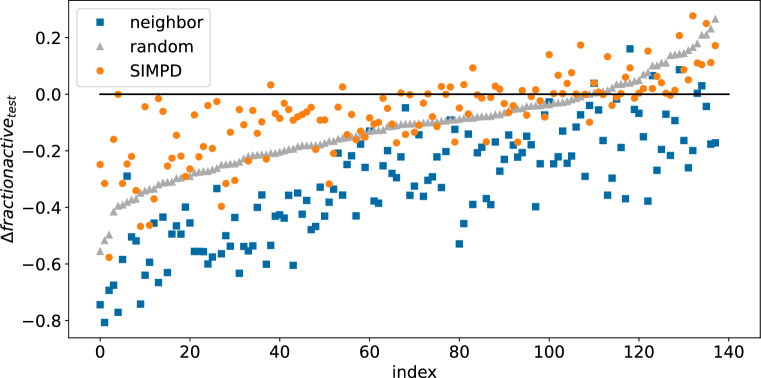
$\Delta \text {frac}_{\text {active}}$(test) values for the random (gray triangles), neighbor (blue squares), and SIMPD (orange circles) splits. The $\Delta$ values were computed relative to the temporal split. The points are ordered by increasing random $\Delta \text {frac}_{\text {active}}$(test)

Figure 5 shows the difference in imbalance ratios in the test set between random, neighbor, and SIMPD splits compared to the temporal splits. Given that this was one of the objectives used in the SIMPD optimization, it is not surprising that the SIMPD values are consistently closer to those from temporal splits than the value with the other two splitting strategies.

**Fig. 6:**
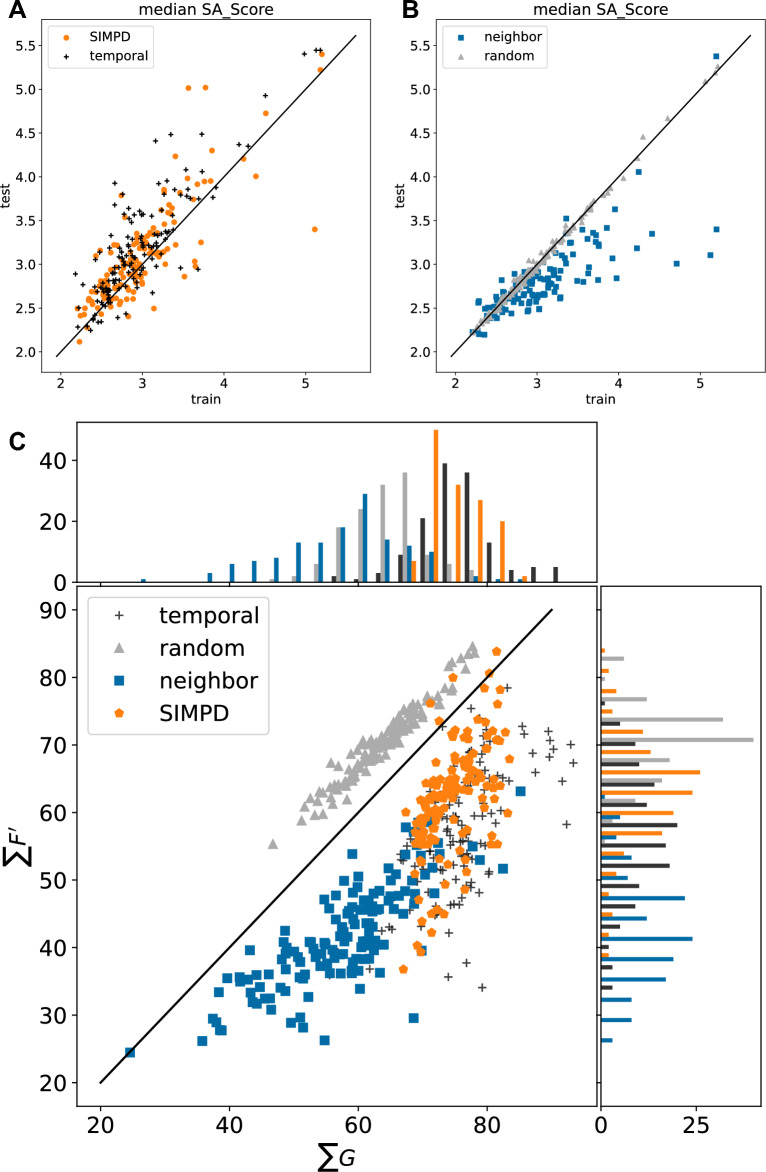
(Top): Comparison of the median SA_Score values in the test and training sets for the four different splitting strategies. The plot is divided into two parts for clarity. **A**: temporal (black crosses) and SIMPD splits (orange circles). **B**: neighbor (blue squares) and random splits (gray triangles). (Bottom, **C**: Spatial statistics summary plot $\sum G$ against $\sum F'$ for the NIBR medicinal chemistry project data sets with temporal (black crosses), random (gray triangles), neighbor (blue squares), and SIMPD (orange circles) training/test splits

Figure 6A, B shows the differences in the median SA_Score values of the training and test sets for each of the project data sets. As discussed above, the median SA_Score is larger in the temporal test set than the training set for most of the data sets. The SIMPD splits reproduce this well. The random and neighbor splits, on the other hand, do not take descriptor values into account and thus, show completely different behaviors. As expected for points that are drawn randomly from a distribution, the random splits show no statistically significant difference (as measured by a Wilcoxon signed-rank test) between the training and test values. Given that computed property values were not used when introducing the neighbor splits, their impact on descriptor values is not known *a priori*. For the NIBR data sets, the neighbor splits tend to have lower median SA_Score values in the test set than the training set. Similar plots for the other three descriptors used in SIMPD, all behaving similarly to SA_Score, are provided in Additional file 1: Fig. S10.

Figure 6C shows that the SIMPD splits have $\sum G$ values closer to those from the temporal splits than either random or neighbor splits. The $\sum F'$ values (*y*-axis of Fig. 6C) for the SIMPD splits tend to fall between random and neighbor splits (like the temporal splits). As discussed above, the temporal, random, and neighbor splits are generally in separate regions of the plot with both the temporal and random sets below the diagonal and slightly overlapping. The splits from SIMPD are almost all located below the diagonal and are shifted towards high $\sum G$, indicating that test-set compounds tend to have at least one quite similar neighbor in the test set. This is in excellent agreement with the temporal splits.

#### Machine-learning performance

**Fig. 7:**
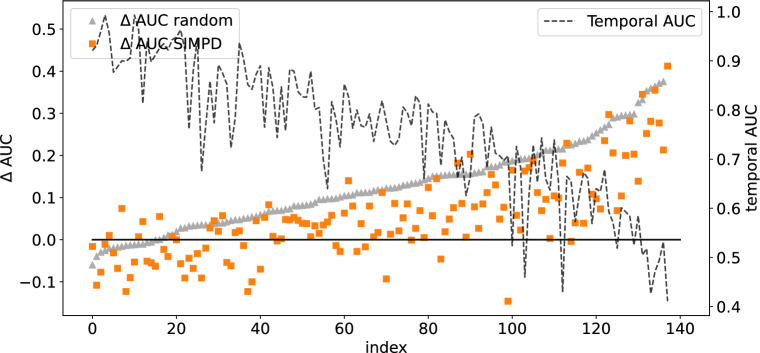
Validation performance of random forest models on the NIBR medicinal chemistry project data sets. The black dashed line and right hand *y*-axis show the AUC values for the temporal splits. The gray triangles and orange circles show $\Delta$AUC values (left hand *y*-axis) for the random and SIMPD splits, respectively. The points are ordered by increasing random $\Delta$AUC

The goal of SIMPD is to produce training/test splits for machine learning which are more similar to the temporal splits observed in medicinal chemistry project data than random or neighbor splits. We have shown above that the SIMPD sets reproduce the differences in descriptors and spatial statistics observed in temporal splits. An additional validation that SIMPD is working as intended is to compare the performance of ML models built and tested using the non-temporal splits with the performance of models built using temporal splits. As can be seen in Fig. 7, the SIMPD splits – like the random splits – tend to be overly optimistic and yield AUC values that are larger than those seen for the temporal splits. The performance of the SIMPD splits is, however, generally closer to the temporal performance than the random splits. To visualize the differences relative to random splits more clearly, results for the neighbor splits were not included in Fig. 7. A plot which includes these values is shown in Additional file 1: Fig. S5.

We also evaluated the performance of the models using Cohen’s $\kappa$, F1 score, and balanced accuracy and the numeric results (along with those for AUC) are in the Additional file 2. Plots analogous to Fig. 7 for Cohen’s kappa and F1 score can be found in Additional file 1: Figs. S7 and S8.

**Fig. 8:**
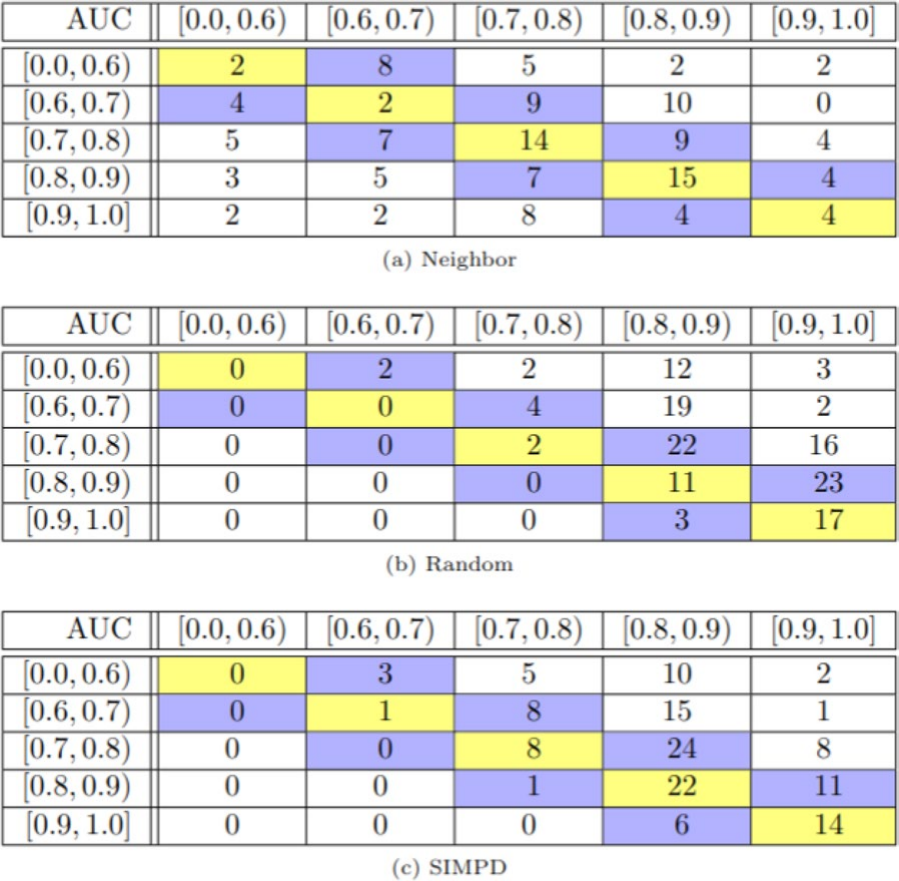
Confusion matrices for the performance of the different splits based on binned AUC values. In each matrix, the rows correspond to the temporal splits while the columns refer to the neighbor (**a**), random (**b**), or SIMPD (**c**) splits

Another way of looking at the ML results is to group the AUC values into bins and then construct “confusion matrices” comparing the AUC values of the neighbor, random, and SIMPD splits with those of the temporal splits (Fig. 8). Here, we see the same trends as discussed above: Neighbor splits tend to be overly pessimistic compared to temporal splits (i.e., larger values below the diagonal in the matrices), while random splits tend to be overly optimistic (i.e., larger values above the diagonal). The results for the SIMPD splits are, again, more similar to random than neighbor, but they are significantly closer to the diagonal than the random splits. This further supports the conclusion that the ML performance from the SIMPD splits is more predictive of the temporal split performance than either random or neighbor splits.

### Applying SIMPD to public data sets

**Fig. 9:**
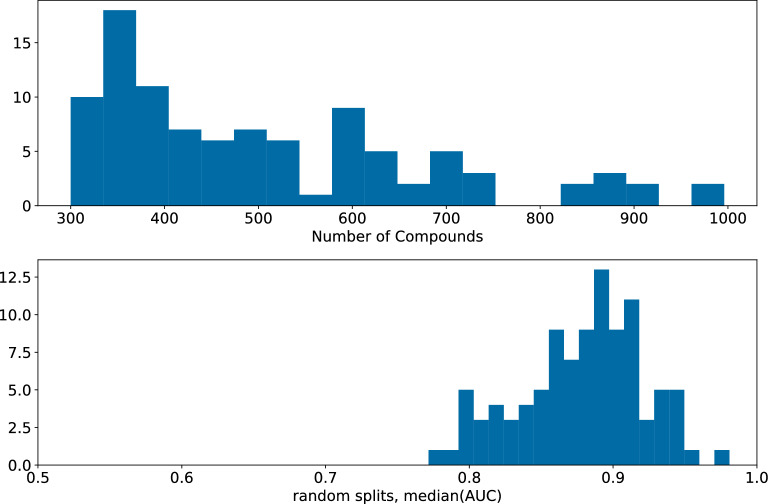
Summary of the 99 data sets extracted from ChEMBL32 (Top): Number of compounds in each data set. (Bottom): Distribution of median AUC values for random forest models built using MFP2 and random splitting for these data sets

Summaries of the number of compounds in each of the 99 ChEMBL32 K$_\text {i}$ data sets along with the median AUC values from random forests built and tested using Morgan fingerprints with radius 2 (MFP2) and random splitting are shown in Fig. 9. The models for the vast majority of the assays have an AUC $>0.8$. This type of high performance is, in our experience, fairly typical for K$_\text {i}$ models built and tested using random splits of ChEMBL data. Although these data sets are slightly more diverse than the NIBR project data sets (see Additional file 1: Fig. S12 for a comparison of the numbers of clusters found in both collections of data sets) and are not small (each data set has at least 300 points), random splits are still too “easy” for use in validating ML models.

**Fig. 10:**
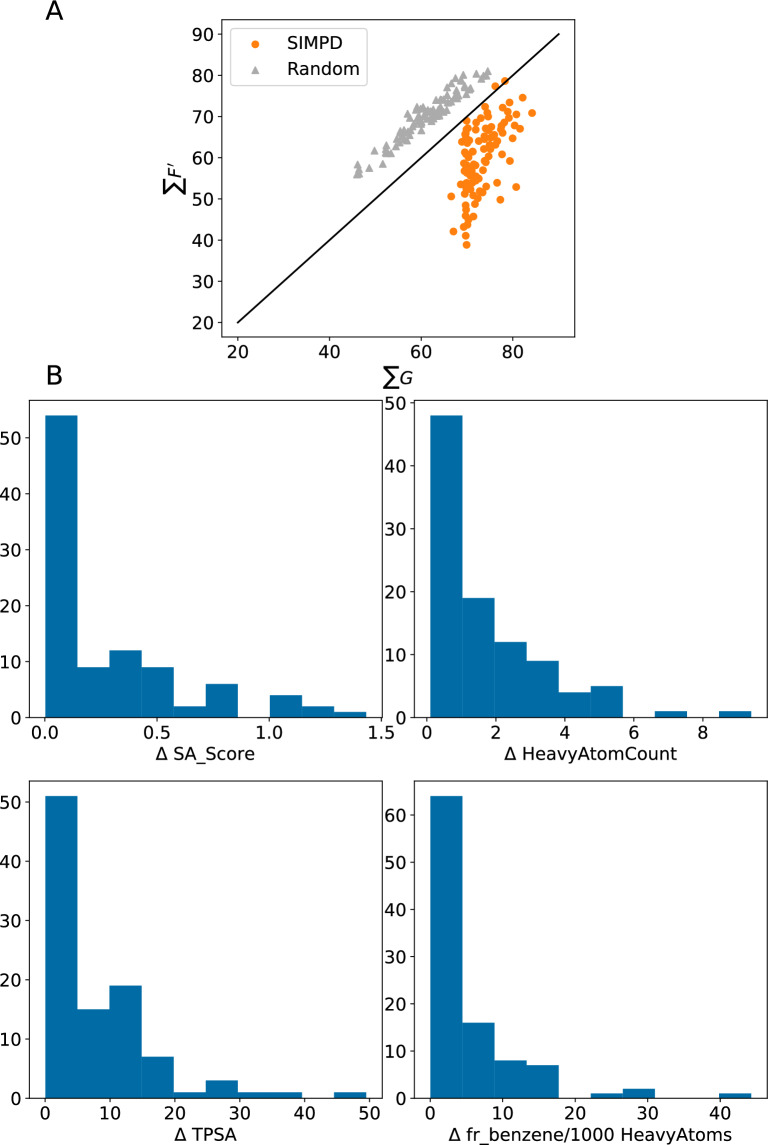
**A**: Spatial statistics summary plot $\sum G$ against $\sum F'$ for the 99 CHEMBL32 data sets with both random (gray triangles) and SIMPD (orange circles) training/test splits. The objectives used in the GA were $10< \sum G - \sum F' < 30$ and $\sum G > 70$. **B**: Histograms of the deviations in the observed training-test descriptor differences from their target values for the SIMPD splits of the 99 ChEMBL32 data sets. The objective used by the MOGA for each of these descriptors was 0

Figure 10A show spatial statistics values for the top solutions from the weighted scoring scheme for each data set. The corresponding values for the four descriptors are given in Fig. 10B. The MOGA was able to do a very good job of satisfying the spatial statistics objectives (compare the orange circles in Fig. 10A with those for the NIBR data sets in Fig. 6C) and a reasonable job of satisfying the descriptor objectives, i.e., the values in the histograms are close to zero.

**Fig. 11:**
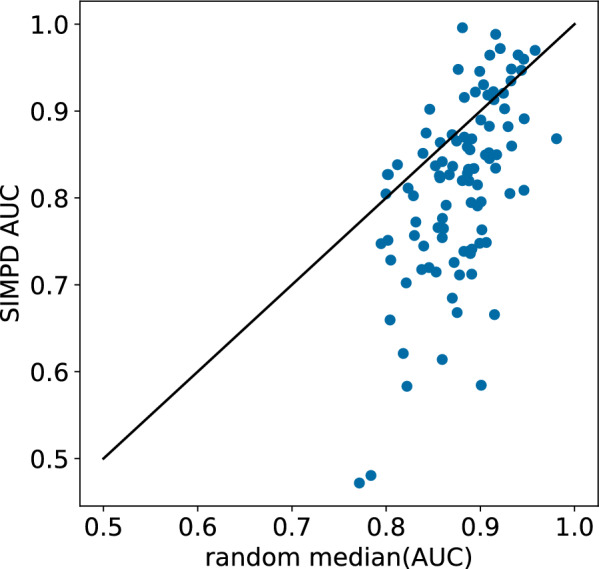
Comparison of ML model performance for SIMPD and random splits of the 99 ChEMBL32 data sets

Finally, we compared the ML performance of the SIMPD splits with random splits for the ChEMBL32 data sets (Fig. 11). As we observed for the NIBR project data sets, the SIMPD splits generally show lower AUC values than random splits. Note that SIMPD is sensitive to initialization (selection of the starting population, stochastic process of the MOGA). When applying the algorithm for real-world model validation, it may be worthwhile use multiple SIMPD splits of each data set. We plan to explore this in the future.

## Conclusion

Our primary goal in this study was to develop an approach to generate training/test splits of data sets, which mimic the differences observed in temporal splits of medicinal chemistry lead-optimization project data better than standard random or neighbor splits. The initial analysis on temporal splits of 138 NIBR medicinal chemistry project data sets allowed us to identify a number of consistent differences in the descriptor distributions and spatial statistics between the early compounds in a project (training set) and the later compounds (test set). Applying our SIMPD algorithm to the project data sets yielded simulated time splits that reproduced these descriptor and spatial statistics differences. We also demonstrated that the SIMPD splits are better at predicting the performance of standard ML models validated on time splits than either random or neighbor splits do.

It is natural to ask whether or not it is possible to avoid the complexity of the SIMPD approach and estimate temporal performance by simply averaging the performance of random splits (which tend to be overly optimistic) and neighbor splits (which tend to be overly pessimistic). We had tested this approach and seen that it does, indeed, lead to error estimates which tend to be closer to the temporal splits than either of the starting points (Additional file 1: Fig. S9). We opted not to further pursue this since the approach is purely *ad hoc* and has no statistical justification.

The curation of high-quality public data sets that resemble the data available in industrial medicinal chemistry projects is a time-consuming and challenging task. In our opinion, SIMPD will be a useful tool for this as it allows us to split a set of compounds into training and test sets which differ in ways similar to what is observed in real project data. However, realistic sets of compounds are needed for this. The collection of 99 ChEMBL32 data sets presented here with training/test splits from SIMPD is a good starting point and a useful complement to or replacement for standard public validation sets. Nevertheless, we intend to continue working to create additional starting points for SIMPD in order to have a broader selection of simulated time-split data sets for use in benchmarking and validating new ML approaches which are intended for use on lead-optimization type problems.

### Supplementary Information

**Additional file 1.** Additional tables and figures.

**Additional file 2.** **project_data_summary.txt**: Summary statistics – e.g. size, active/inactive counts, spatial statistics values, median descriptor values, etc. – calculated for the various splits of the NIBR project data sets. **project_data_ML_summary.txt**: Summary statistics for ML performance across the various splits of the NIBR project data sets. **project_data_baselineML_summary.txt**: Summary statistics for the baseline ML performance across the various splits of the NIBR project data sets. **full_chembl_assay_list.csv**: Details of the 99 ChEMBL32 data sets.

## Data Availability

The software for creating, optimizing, and evaluating simulated molecular time series based upon collections of molecules and data is available as open-source Python code at github.com/rinikerlab/molecular_time_series. This package includes the Jupyter notebooks and Python scripts used to generate the data sets discussed here from a local installation of the ChEMBL database. The 99 SIMPD data sets created from ChEMBL32 are available in the GitHub repository, github.com/rinikerlab/molecular_time_series.
