# Supplementary material for: SIMPD: an algorithm for generating simulated time splits for validating machine learning approaches
Source: J Cheminform. 2023 Dec 11;15:119. doi: 10.1186/s13321-023-00787-9 (PMC10712068; doi:10.1186/s13321-023-00787-9)
Supplement: Supplementary file 1 — Additional file 1. Additional tables and figures. [file 13321_2023_787_MOESM1_ESM.pdf]

ADDITIONAL FILE 1
SIMPD: an Algorithm for Generating Simulated Time Splits for Validating Machine Learning Approaches

Gregory A. Landrum,*[a] Maximilian Beckers,[b] Jessica Lanini,[b], Nadine Schneider,*[b] Nikolaus Stiefl,[b] and Sereina Riniker*[a]

[a] *Department of Chemistry and Applied Biosciences, ETH Zürich, Vladimir-Prelog-Weg 2, 8093 Zürich, Switzerland. E-mail: glandrum@ethz.ch, sriniker@ethz.ch*

[b] *Novartis Institutes for BioMedical Research, Novartis Pharma AG, Novartis Campus, 4002 Basel, Switzerland.*

1 Additional Tables

Table S1: Summary of the distribution changes for the descriptors studied. The meanings of the columns are: **Sign** = sign of the difference between training and test; **Frac** = fraction of the data sets showing a change with that sign; **Median(frac change)** = median fractional change of the value across the data sets; **Median(train)** = median value of the property in the training set; **Number of projects** = number of projects where the difference in the training/test distributions was statistically significant.

Property	Sign	Frac	Median (frac change)	Median (train)	Number of projects
SA_Score	1	0.88	0.09	2.84	109
Chi2n	1	0.76	0.11	7.83	119
Chi3n	1	0.80	0.14	5.57	112
Chi0n	1	0.76	0.10	17.39	117
NumValenceElectrons	1	0.77	0.10	162.00	116
Chi0v	1	0.77	0.09	17.80	115
Chi1n	1	0.77	0.10	10.09	114
BCUT2D_CHGHI	1	0.81	0.02	2.25	109
Chi0	1	0.76	0.10	22.04	115
LabuteASA	1	0.76	0.08	181.98	114
Chi1	1	0.77	0.09	15.06	113
HeavyAtomCount	1	0.75	0.09	31.00	114
Ipc	1	0.77	2.05	13007916.85	112
Ipc/1000 HeavyAtoms	1	0.77	1.80	426443158.32	112
BCUT2D_LOGPLOW/1000 HeavyAtoms	1	0.77	0.07	-74.33	112
Chi4n	1	0.80	0.15	3.79	108
Kappa1	1	0.76	0.10	21.30	112
EState_VSA2	1	0.78	0.43	12.33	109
BCUT2D_LOGPHI	1	0.74	0.01	2.31	113
HeavyAtomMolWt	1	0.76	0.09	415.51	110
SlogP_VSA2	1	0.79	0.24	40.65	107

BCUT2D_CHGLO/1000 HeavyAtoms	1	0.72	0.07	-71.44	115
Chi2v	1	0.75	0.10	8.35	110
TPSA	1	0.76	0.14	88.61	109
MolWt	1	0.76	0.09	442.47	109
ExactMolWt	1	0.76	0.09	442.09	109
BertzCT	1	0.73	0.10	1116.01	112
SlogP_VSA5	1	0.76	0.29	29.50	106
FractionCSP3	1	0.77	0.23	0.32	105
Chi4v	1	0.78	0.14	4.16	104
Chi3v	1	0.79	0.13	5.93	102
Chi1v	1	0.75	0.10	10.61	107
MolMR	1	0.71	0.09	119.28	112
Chi4n/1000 HeavyAtoms	1	0.71	0.07	123.65	110
Chi3n/1000 HeavyAtoms	1	0.72	0.06	176.89	108
Chi2n/1000 HeavyAtoms	1	0.69	0.04	250.95	111
Kappa2	1	0.72	0.09	8.71	105
EState_VSA2/1000 HeavyAtoms	1	0.73	0.32	454.10	103
SlogP_VSA2/1000 HeavyAtoms	1	0.78	0.15	1309.74	96
PEOE_VSA8	1	0.69	0.25	29.32	108
SMR_VSA5	1	0.76	0.32	26.19	97
NumHeteroatoms	1	0.66	0.12	8.00	111
SlogP_VSA5/1000 HeavyAtoms	1	0.71	0.25	873.77	103
MinPartialCharge/1000 HeavyAtoms	1	0.75	0.10	-13.24	97
FpDensityMorgan1	1	0.71	0.05	1.09	101
VSA_EState2	1	0.71	0.20	19.69	101
FpDensityMorgan2	1	0.66	0.04	1.86	107
SMR_VSA5/1000 HeavyAtoms	1	0.71	0.29	867.28	100
MaxEStateIndex	1	0.72	0.03	12.89	99
MaxAbsEStateIndex	1	0.72	0.03	12.89	99
NumValenceElectrons/1000 HeavyAtoms	1	0.72	0.01	5200.00	99
SMR_VSA1	1	0.69	0.35	13.52	101
FractionCSP3/1000 HeavyAtoms	1	0.71	0.20	9.83	99
NumAliphaticRings/1000 HeavyAtoms	1	0.65	0.22	30.30	106
Kappa3	1	0.66	0.12	4.38	105
NOCount	1	0.69	0.14	7.00	100
Chi2v/1000 HeavyAtoms	1	0.62	0.04	263.64	110
NOCount/1000 HeavyAtoms	1	0.67	0.08	223.61	102
SlogP_VSA3/1000 HeavyAtoms	1	0.63	0.34	303.65	105
TPSA/1000 HeavyAtoms	1	0.63	0.10	2864.17	105
SlogP_VSA4/1000 HeavyAtoms	1	0.62	0.43	190.90	105
Chi4v/1000 HeavyAtoms	1	0.63	0.07	129.17	103
SlogP_VSA3	1	0.64	0.44	9.78	102
VSA_EState3	1	0.64	0.38	8.06	102
HallKierAlpha/1000 HeavyAtoms	1	0.64	0.08	-103.38	102
SA_Score/1000 HeavyAtoms	1	0.64	0.08	92.56	102
Chi3v/1000 HeavyAtoms	1	0.64	0.06	184.09	102
EState_VSA3	1	0.66	0.24	22.54	99
PEOE_VSA1	1	0.65	0.26	14.99	99
NumHeteroatoms/1000 HeavyAtoms	1	0.65	0.09	259.26	99
EState_VSA10/1000 HeavyAtoms	1	0.62	0.28	313.02	102
SMR_VSA3/1000 HeavyAtoms	1	0.63	0.18	519.11	100
PEOE_VSA7	1	0.64	0.16	43.49	99

VSA_EState1	1	0.69	0.56	10.44	91
PEOE_VSA10	1	0.60	0.33	6.61	103
NumSaturatedRings/1000 HeavyAtoms	1	0.61	0.25	28.57	102
SMR_VSA3	1	0.62	0.22	15.11	100
Chi1n/1000 HeavyAtoms	1	0.63	0.03	326.11	99
NumHAcceptors/1000 HeavyAtoms	1	0.62	0.13	181.82	99
MinAbsPartialCharge	1	0.63	0.08	0.26	97
VSA_EState7	1	0.61	0.49	2.84	99
SMR_VSA1/1000 HeavyAtoms	1	0.64	0.25	420.81	94
SMR_VSA6	1	0.65	0.28	18.41	92
MaxPartialCharge	1	0.60	0.07	0.26	98
VSA_EState1/1000 HeavyAtoms	1	0.61	0.51	334.14	97
PEOE_VSA8/1000 HeavyAtoms	1	0.62	0.20	977.01	95
PEOE_VSA9	1	0.64	0.32	16.13	90
VSA_EState8	1	0.60	0.39	3.33	91
MinPartialCharge	1	0.62	0.07	-0.40	87
MolLogP	-1	0.61	0.15	3.77	93
LabuteASA/1000 HeavyAtoms	-1	0.61	0.01	5850.72	95
EState_VSA6/1000 HeavyAtoms	-1	0.67	0.36	525.37	90
EState_VSA7	-1	0.62	0.35	24.27	99
MinEStateIndex/1000 HeavyAtoms	-1	0.62	0.55	-16.23	100
MinAbsEStateIndex	-1	0.67	0.43	0.10	92
MaxPartialCharge/1000 HeavyAtoms	-1	0.60	0.11	8.86	105
Kappa2/1000 HeavyAtoms	-1	0.64	0.04	277.58	99
VSA_EState5	-1	0.62	1.00	0.38	104
SMR_VSA10/1000 HeavyAtoms	-1	0.63	0.21	769.31	101
SlogP_VSA1/1000 HeavyAtoms	-1	0.66	0.20	332.30	97
Kappa3/1000 HeavyAtoms	-1	0.61	0.07	141.03	107
VSA_EState5/1000 HeavyAtoms	-1	0.63	0.94	10.23	103
SMR_VSA7	-1	0.63	0.10	60.62	108
HallKierAlpha	-1	0.65	0.09	-3.17	104
MaxEStateIndex/1000 HeavyAtoms	-1	0.67	0.09	390.80	102
MaxAbsEStateIndex/1000 HeavyAtoms	-1	0.67	0.09	390.80	102
Chi1/1000 HeavyAtoms	-1	0.69	0.00	481.85	98
MinAbsEStateIndex/1000 HeavyAtoms	-1	0.69	0.45	3.01	98
PEOE_VSA6	-1	0.69	0.33	23.73	98
qed	-1	0.64	0.14	0.54	108
MolMR/1000 HeavyAtoms	-1	0.70	0.02	3849.45	100
BalabanJ	-1	0.70	0.07	1.60	103
EState_VSA7/1000 HeavyAtoms	-1	0.71	0.34	774.57	102
BCUT2D_MRLOW/1000 HeavyAtoms	-1	0.66	0.37	2.02	111
MinEStateIndex	-1	0.71	0.41	-0.53	103
SlogP_VSA6	-1	0.71	0.15	47.39	103
FpDensityMorgan1/1000 HeavyAtoms	-1	0.71	0.10	34.45	104
MolLogP/1000 HeavyAtoms	-1	0.72	0.14	124.73	103
BCUT2D_MRLOW	-1	0.70	0.39	0.07	109
FpDensityMorgan2/1000 HeavyAtoms	-1	0.70	0.10	59.24	108
MaxAbsPartialCharge/1000 HeavyAtoms	-1	0.76	0.10	13.29	100
BCUT2D_MWHI/1000 HeavyAtoms	-1	0.71	0.14	650.71	109
NumAromaticRings/1000 HeavyAtoms	-1	0.71	0.13	100.00	109
VSA_EState6	-1	0.74	0.25	10.65	106
BCUT2D_MWLOW	-1	0.74	0.01	10.02	105

qed/1000 HeavyAtoms	-1	0.74	0.23	17.07	107
PEOE_VSA6/1000 HeavyAtoms	-1	0.78	0.31	742.60	101
BCUT2D_LOGPLOW	-1	0.78	0.03	-2.36	103
BCUT2D_CHGLO	-1	0.72	0.02	-2.26	112
FpDensityMorgan3/1000 HeavyAtoms	-1	0.72	0.10	81.60	112
BCUT2D_CHGHI/1000 HeavyAtoms	-1	0.74	0.08	72.07	109
SMR_VSA7/1000 HeavyAtoms	-1	0.75	0.15	1938.97	110
BalabanJ/1000 HeavyAtoms	-1	0.78	0.15	51.12	107
BCUT2D_LOGPHI/1000 HeavyAtoms	-1	0.77	0.08	74.65	115
BCUT2D_MWLOW/1000 HeavyAtoms	-1	0.77	0.09	320.13	114
VSA_EState6/1000 HeavyAtoms	-1	0.80	0.25	359.27	110
BCUT2D_MRHI/1000 HeavyAtoms	-1	0.80	0.10	202.84	113
SlogP_VSA6/1000 HeavyAtoms	-1	0.82	0.17	1565.12	114
NumAromaticCarbocycles/1000 HeavyAtoms	-1	0.80	0.19	43.48	117
fr_benzene/1000 HeavyAtoms	-1	0.81	0.19	43.48	118

2 Additional Figures

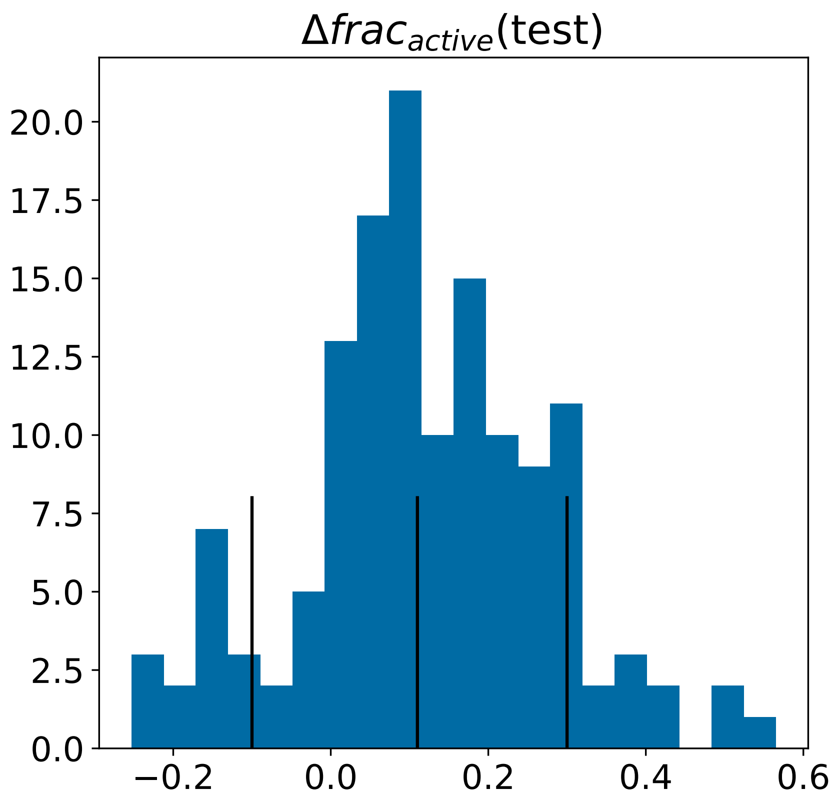

Figure S1: Distribution of $\Delta\text{frac}_{\text{active}}$(test) values for the temporal splits of the 138 NIBR medicinal chemistry project data sets.

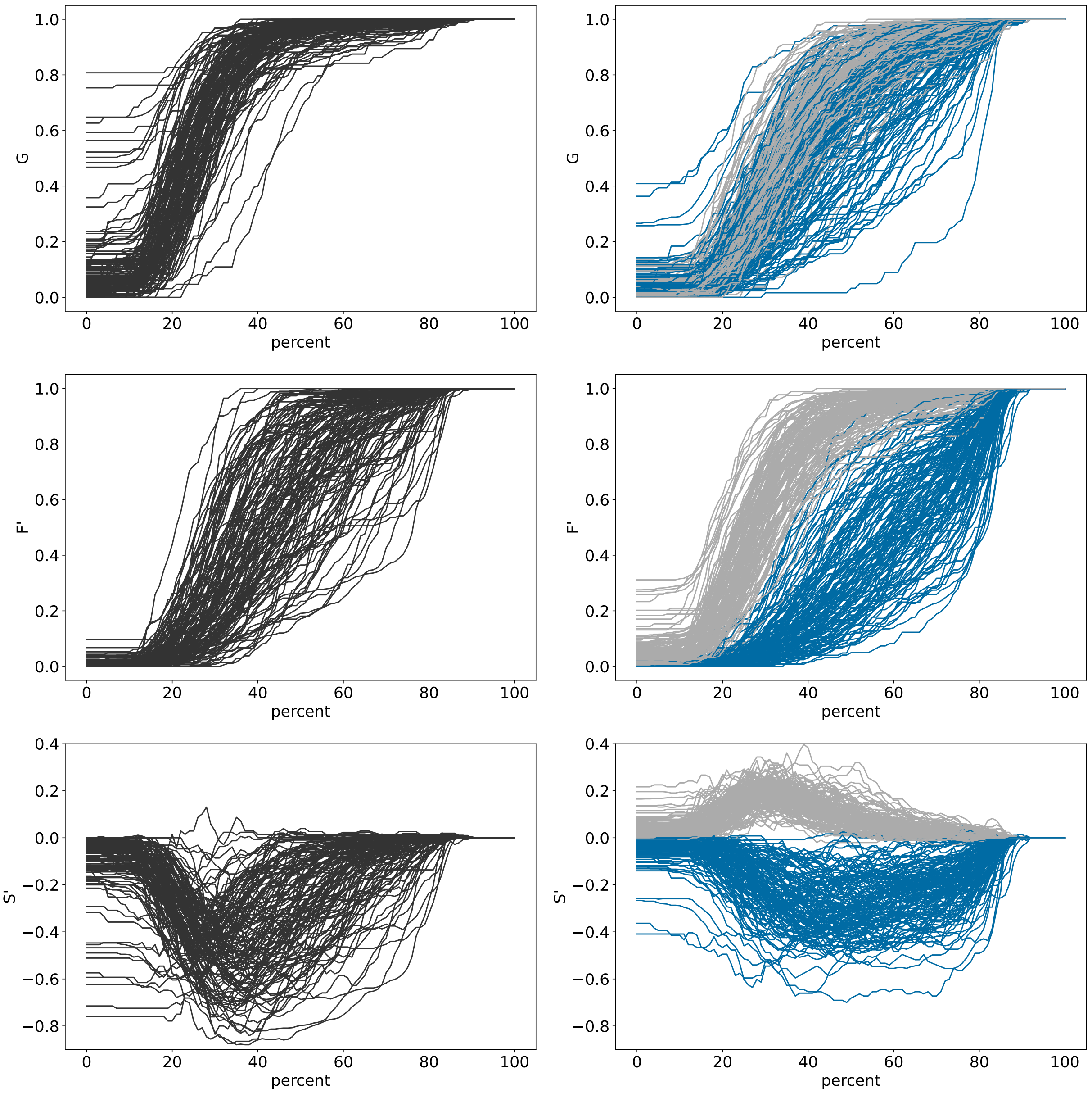

Figure S2: Spatial statistics curves for the NIBR medicinal chemistry project data sets. In each row the left plot shows the values for the temporal splits while the right plot shows the values for the random (gray line) or neighbor (blue line) splits. **First row**: G as a function of the percentage of the data set. G is the CDF for distances between test-set compounds and their nearest neighbors in the test set. **Second row**: F' as a function of the percentage of the data set. F' is the CDF for distances between test-set compounds and their nearest neighbors in the training set. **Third row**: S' as a function of the percentage of the data set. S' is defined to be $G - F'$.

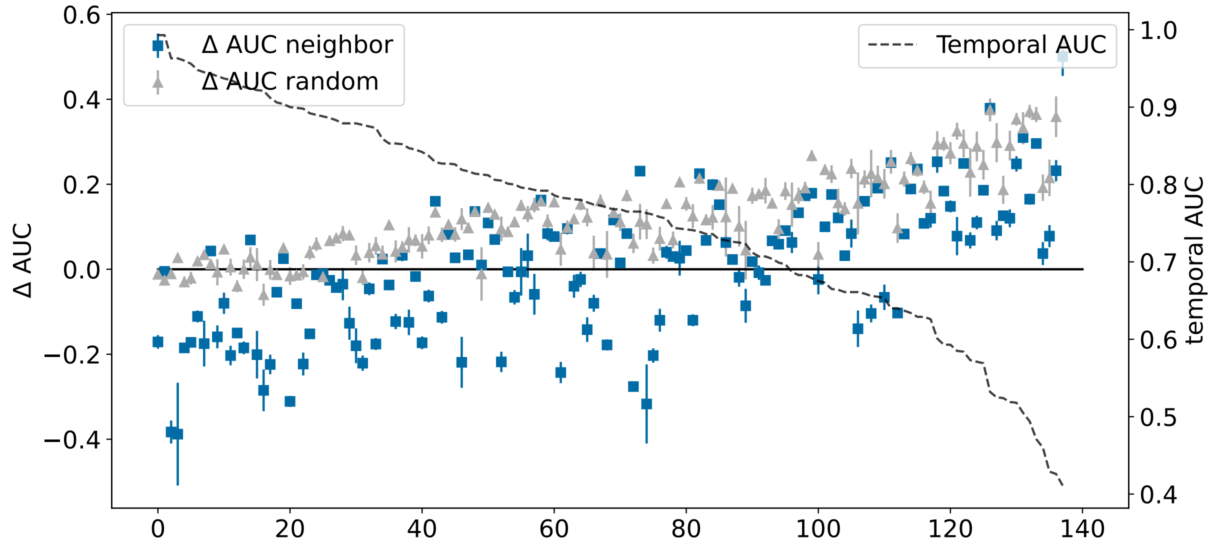

Figure S3: Validation performance of random forest models on the NIBR project data sets. The black dashed line and right hand y-axis show the AUC values for the temporal splits. The gray triangles and blue squares show ΔAUC values for the random and neighbor splits, respectively. The error bars show standard deviations. The points are ordered by decreasing temporal AUC values.

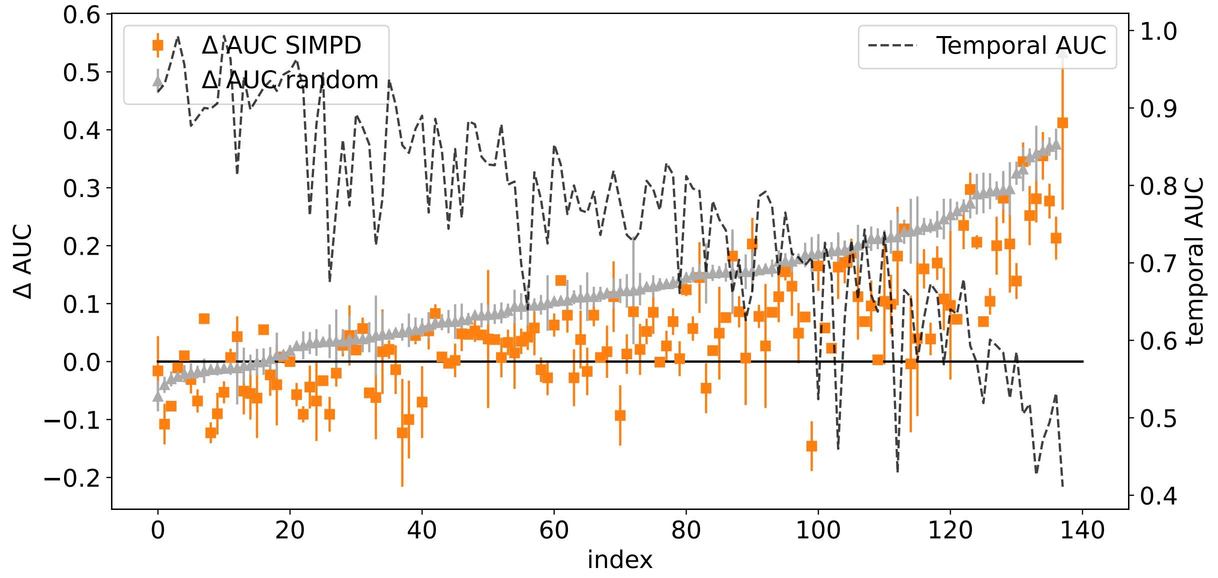

Figure S4: Validation performance of random forest models on the NIBR project data sets. The black dashed line and right hand y-axis show the AUC values for the temporal splits. The gray triangles and orange circles show ΔAUC values for the random and SIMPD splits, respectively. The error bars show standard deviations. The points are ordered by increasing random ΔAUC.

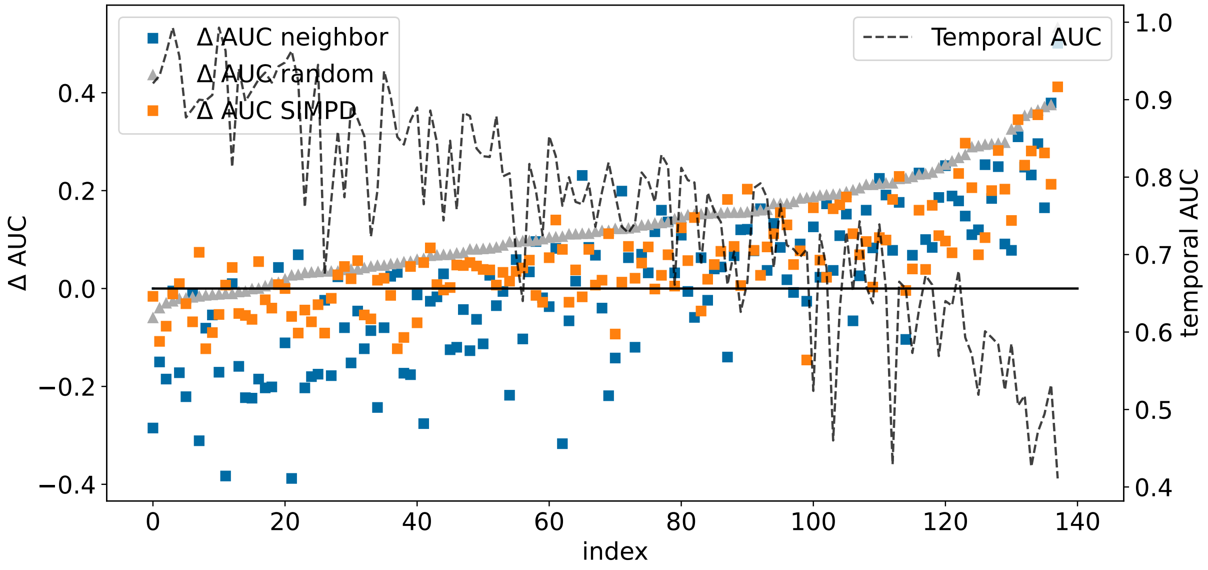

Figure S5: Validation performance of random forest models on the NIBR project data sets. The black dashed line and right hand *y*-axis show the AUC values for the temporal splits. The gray triangles, blue squares, and orange circles show ΔAUC values for the random, neighbor, and SIMPD splits, respectively. The points are ordered by increasing random ΔAUC.

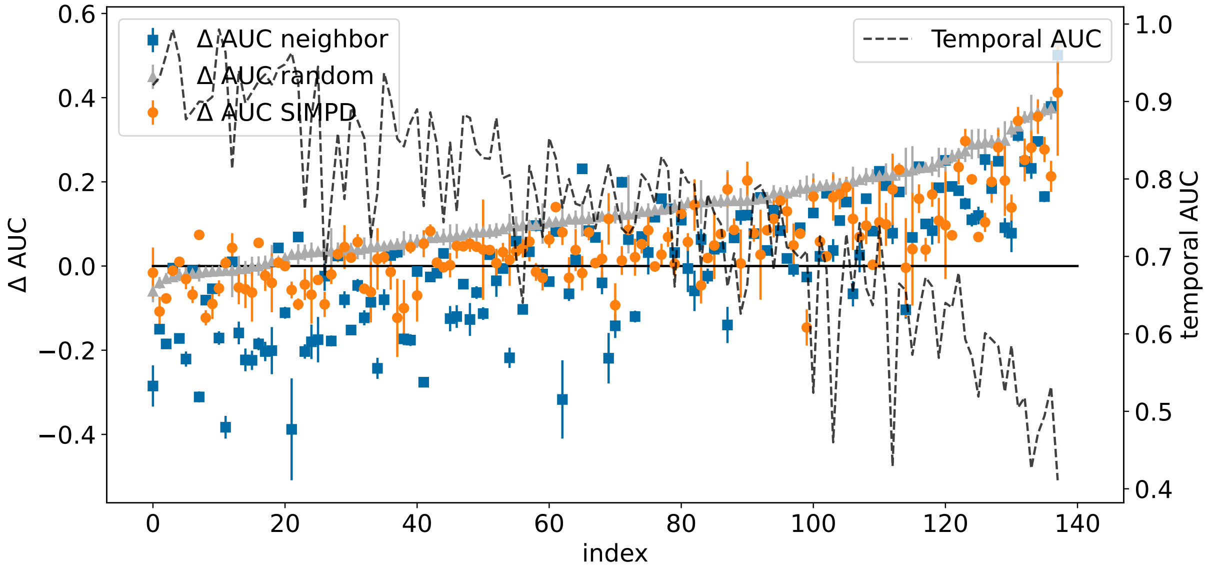

Figure S6: Same data as in Figure S5 but including error bars, which show standard deviations.

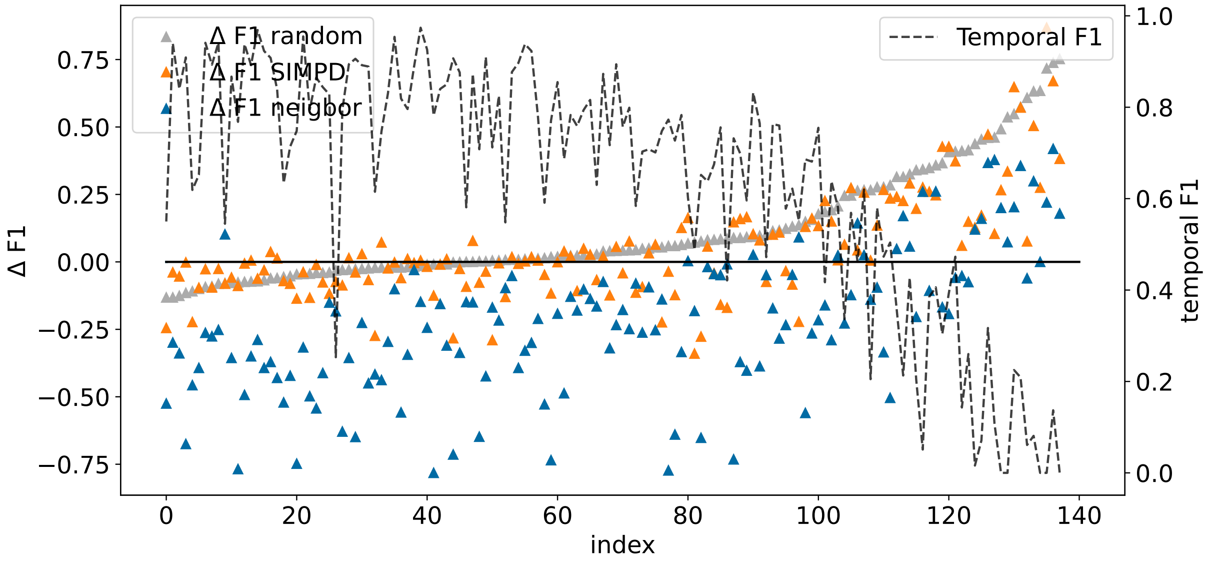

Figure S7: Validation performance of random forest models on the NIBR project data sets. The black dashed line and right hand *y*-axis show the F1 score values for the temporal splits. The gray triangles, blue squares, and orange circles show ΔF1 values for the random, neighbor, and SIMPD splits, respectively. The points are ordered by increasing random ΔF1.

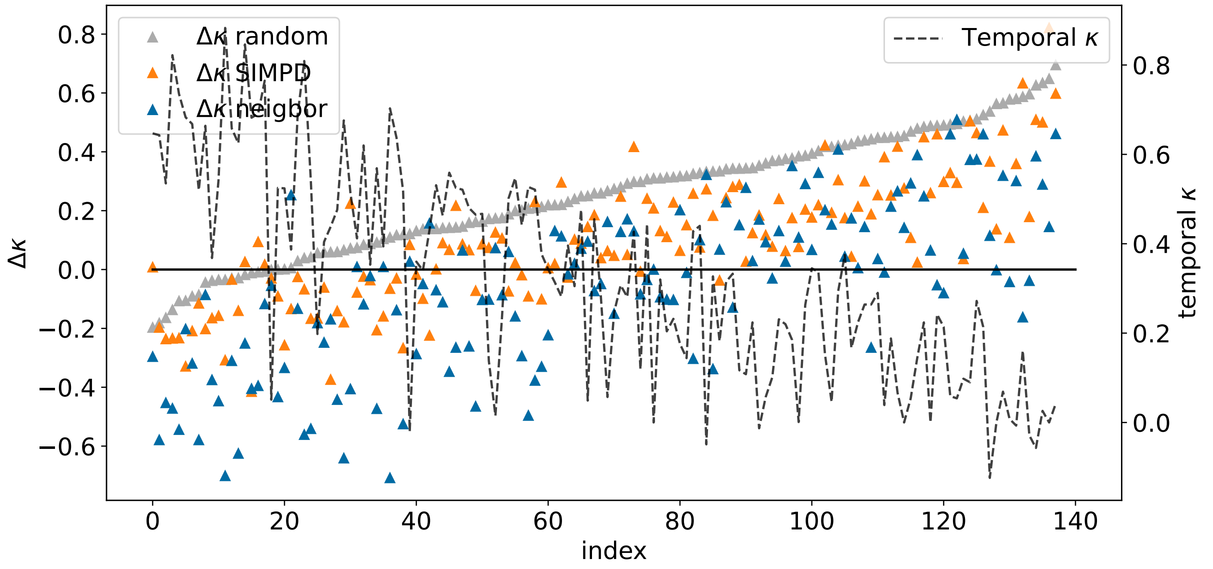

Figure S8: Validation performance of random forest models on the NIBR project data sets. The black dashed line and right hand *y*-axis show the Cohen's κ score values for the temporal splits. The gray triangles, blue squares, and orange circles show $\Delta\kappa$ values for the random, neighbor, and SIMPD splits, respectively. The points are ordered by increasing random $\Delta\kappa$.

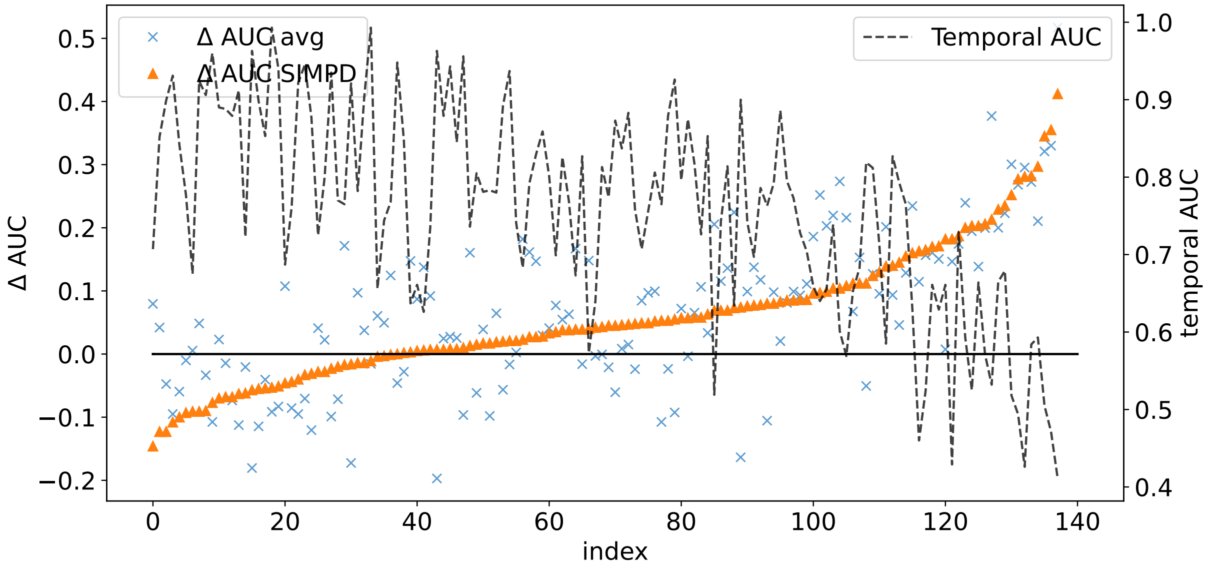

Figure S9: Comparison of SIMPD results with a simple error estimate calculated by averaging the ΔAUROC values for random and neighbor splits. The black dashed line and right hand *y*-axis show the AUROC score values for the temporal splits. The cyan x's and orange circles show ΔAUROC values for the simple error estimate and the SIMPD splits, respectively. The points are ordered by increasing SIMPD ΔAUROC.

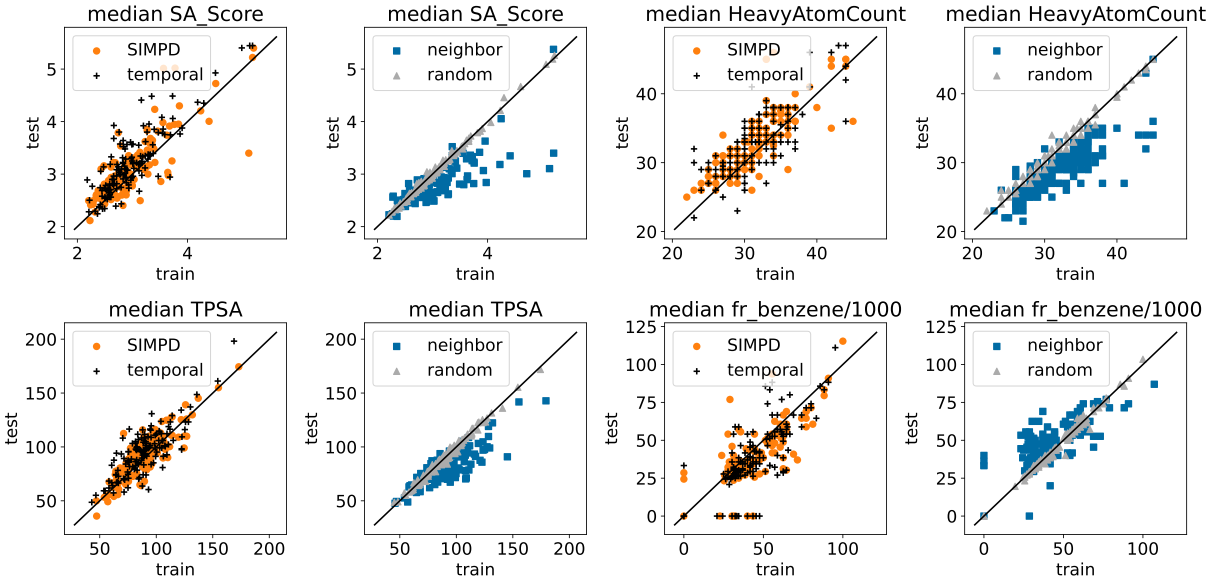

Figure S10: Comparison of the median descriptor values in the training and test sets for the four different splitting strategies. The plot is divided into two parts for clarity. (Left panels): temporal (black crosses) and SIMPD splits (orange circles). (Right panels): neighbor (blue squares) and random splits (gray triangles).

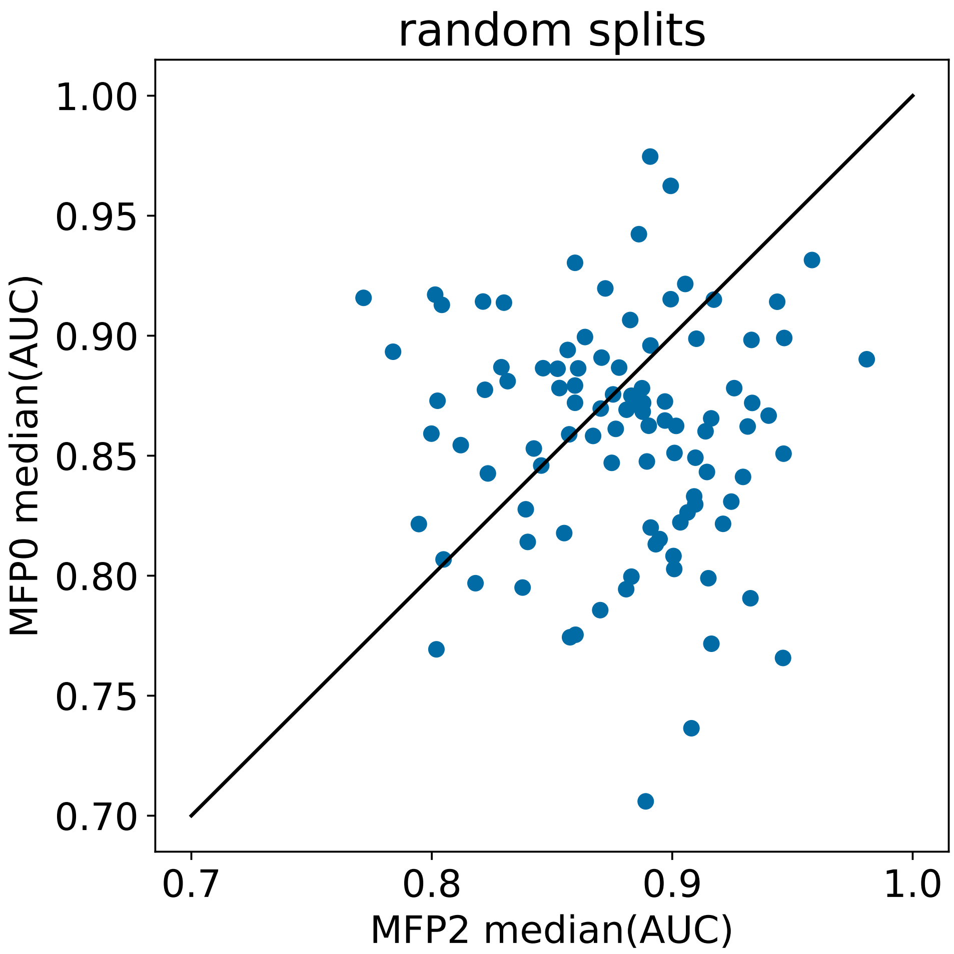

Figure S11: Comparison of the median AUC values for random forest models built on the 99 ChEMBL32 assay data sets with MFP2 fingerprints (x-axis) and MFP0 fingerprints (y-axis).

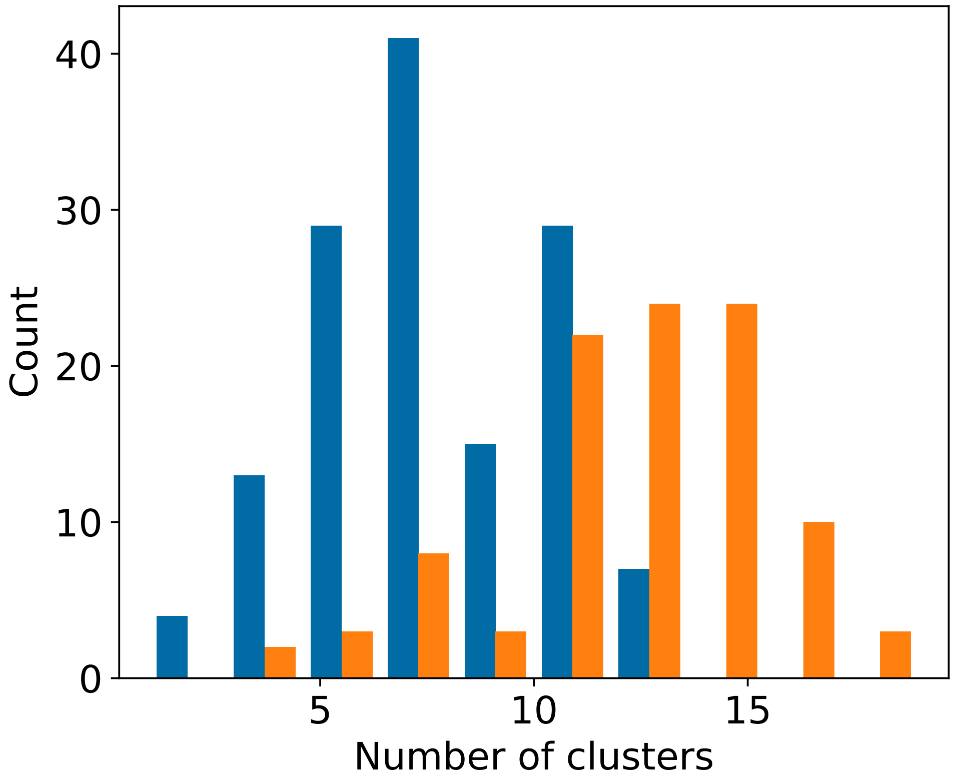

Figure S12: Comparison of the number of starting clusters in the medicinal chemistry project data sets (blue) and the ChEMBL data sets used for the SIMPD algorithm (orange). See the discussion of the SIMPD algorithm for a description of the clustering algorithm used.
